# A Transcriptomic Approach to the Recruitment of Venom Proteins in a Marine Annelid

**DOI:** 10.3390/toxins13020097

**Published:** 2021-01-28

**Authors:** Ana P. Rodrigo, Ana R. Grosso, Pedro V. Baptista, Alexandra R. Fernandes, Pedro M. Costa

**Affiliations:** Applied Molecular Biosciences Unit (UCIBIO), Departamento de Ciências da Vida, Faculdade de Ciências e Tecnologia da Universidade Nova de Lisboa, 2829-516 Caparica, Portugal; ar.grosso@fct.unl.pt (A.R.G.); pmvb@fct.unl.pt (P.V.B.)

**Keywords:** Annelida, marine environment, protein recruitment, selective pressure, toxins, whole-transcriptome sequencing

## Abstract

The growing number of known venomous marine invertebrates indicates that chemical warfare plays an important role in adapting to diversified ecological niches, even though it remains unclear how toxins fit into the evolutionary history of these animals. Our case study, the Polychaeta *Eulalia* sp., is an intertidal predator that secretes toxins. Whole-transcriptome sequencing revealed proteinaceous toxins secreted by cells in the proboscis and delivered by mucus. Toxins and accompanying enzymes promote permeabilization, coagulation impairment and the blocking of the neuromuscular activity of prey upon which the worm feeds by sucking pieces of live flesh. The main neurotoxins (“phyllotoxins”) were found to be cysteine-rich proteins, a class of substances ubiquitous among venomous animals. Some toxins were phylogenetically related to Polychaeta, Mollusca or more ancient groups, such as Cnidaria. Some toxins may have evolved from non-toxin homologs that were recruited without the reduction in molecular mass and increased specificity of other invertebrate toxins. By analyzing the phylogeny of toxin mixtures, we show that Polychaeta is uniquely positioned in the evolution of animal venoms. Indeed, the phylogenetic models of mixed or individual toxins do not follow the expected eumetazoan tree-of-life and highlight that the recruitment of gene products for a role in venom systems is complex.

## 1. Introduction

The recent discovery of the first crustacean venom [1] is compelling evidence for the commonness of chemical warfare amongst marine eumetazoans. It is an addition to already known venomous marine animals, such as cnidarians, cone snails, cephalopods and fish, as well as a recent entry: the Polychaeta [2]. Since such forms of chemical warfare constitute a simple but efficient strategy for survival, it is not surprising to find venoms, which are usually complex cocktails of salts and proteinaceous compounds, in ancient groups. Indeed, addressing the evolution of venoms and the systems involved, understanding how this mixture emerged to provide adaptive leverage, as well as its ecological and morphological role, is paramount [3]. However, and despite the biotechnological potential of conotoxins [4], marine invertebrate toxinology is still lagging behind its terrestrial counterpart.

Assisted by next-generation sequencing, “venomics” enables discovery and molecular characterization of multiple venom proteins and peptides, including in organisms with incipient genomic annotation [2,5,6]. It has already offered an evolutionary insight on the recruitment of protein families into venoms of terrestrial animals [7,8,9], shedding light on the importance of orphan genes, neofunctionalization of duplicates by amino acid substitution and the evolutionary pressures regulating the optimal use of metabolically expensive resources [7,10,11,12]. Indeed, it has been shown that snake venoms evolved from proteins holding non-toxin functions toward the “three-finger toxin” group that includes neurotoxins, anti-coagulants and cytotoxins [7,13]. Accordingly, the first insights into Polychaeta toxins are revealing a wider span of species than anticipated, including glycerids (bloodworms), amphinomids (especially fireworms) and phyllodocids [2,14,15]. Even though information on toxin activity is scarce, the search for homologs suggests a combination of cytotoxins, hemotoxins, neurotoxins and venom-specific proteolytic enzymes [16] that, as for other invertebrates like octopuses, permeabilize the tissue of their prey to facilitate infiltration [17].

Recently, we disclosed that *Eulalia* sp. (Phyllodocidae), a toxin-secreting intertidal predator which is rather inconspicuous besides its bright green color, secretes toxic mucus whose noxious proteins (hitherto termed “phyllotoxins”) assist its uncanny feeding behavior: the worm stuns its prey and sucks a piece of flesh with its powerful proboscis, albeit devoid of jaws and complex glands [15]. It should therefore be noted that phyllotoxins are not injected and therefore the secretions do not really constitute a venom. Also, since no wound is actually inflicted to deliver the noxious substances, as Nelsen et al. [18] have suggested, the novel term “toxungen” is actually more accurate, since the toxins are applied via surface contact whereas poisons, by definition, lack delivery structures altogether. In fact, *Eulalia* possesses specialized tentacles at the tip of the proboscis, equipped with mucocytes and a layer of calix-like cells that, when subjected to pressure, e.g., by direct contact with prey, rapidly release toxins from dense intraplasmatic vesicles [19]. It should be noted that the presence of lytic enzymes in the proboscis has previously been described decades ago [20,21,22], although toxins were not then referred to. We thus disclosed that *Eulalia* secretes a cocktail of toxins that likely includes immobilizers or relaxants and enzymes that partially digest the tissue of preferential prey: mollusks, barnacles and other Polychaeta [15]. Despite many advances in recent years, it should be noted that the Polychaeta are as diverse as they are phylogenetically complex, as well as being seemingly not monophyletic, with many groups lacking clear evolutionary positioning and missing support from molecular systematics [23,24,25]. Similarly, there is little information on the recruitment of protein-encoding genes into the composition of venoms in these protostomes [26,27]. The present work aims at identifying the main transcripts of toxins secreted by *Eulalia* and comparing them to representative homologs among known venomous taxa.

## 2. Results and Discussion

### 2.1. Discovery of Putative Toxins

**Figure 1:**
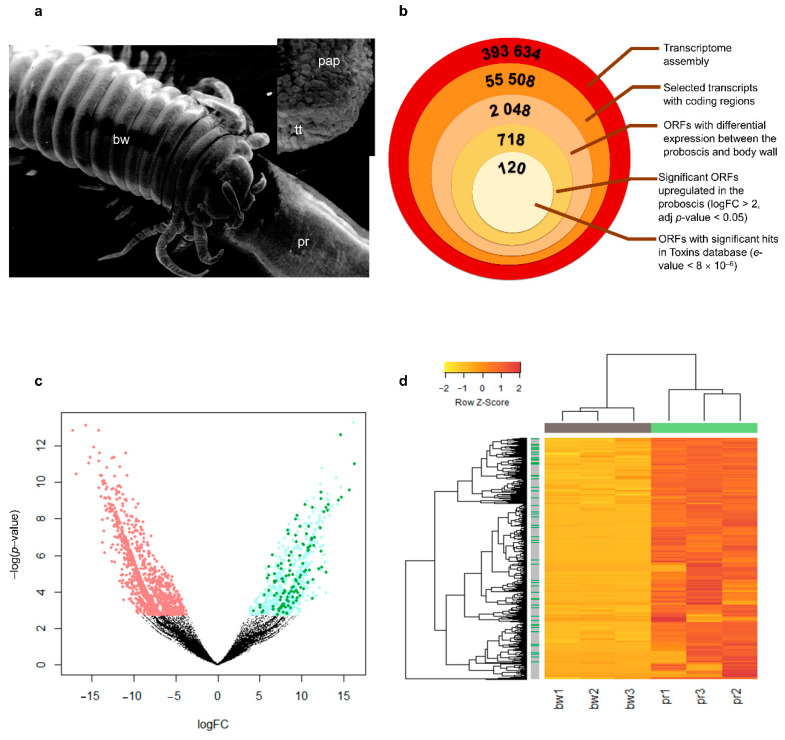
Transcriptomics revealed toxin-like proteins secreted by *Eulalia*’s proboscis. (**a**) Scanning electron microscopy (SEM) micrographs of the head of *Eulalia* evidencing the body wall (bw), which comprises the skin and subjacent musculature, and the everted proboscis (pr), also termed the trunk of eversible pharynx. Inset: tip of the fully everted proboscis, covered with sensorial papillae (pap) and the specialized tentacles around the mouth (tt) that the animal uses to deliver toxin-containing mucus to the surface of prey. (**b**) Tiered whole-transcriptome refinement to select transcripts of interest for toxin identification. (**c**) Volcano plot of open reading frames (ORFs) under- (red) and overexpressed (blue) in the proboscis (logFC > 2) and, from those, the ones with hits in the Toxins database (green). (**d**) Heatmap showing the 718 ORFs that yielded overexpression in the proboscis (pr), compared with the body wall (bw), for three different worms (identified as 1, 2 and 3). Side bar (grey) is indicative of genes with a match in the Toxins database (green). Complete linkage is employed as a clustering function and Euclidian distances as the metric. Data are row-normalized.

The proboscis of *Eulalia* is an eversible pharynx with multiple roles, particularly sensing and feeding. Our previous work described the structure and role of the organ in feeding, which showed the existence of specialized tentacles in its tip that are used for the delivery of noxious substances produced in specialized calix cells lining the interior epithelium of the organ [13,17]. Consequently, we hypothesized that putative toxin mRNAs could be identified by running RNA-seq in the proboscis (pr) and body wall (bw) to pinpoint overexpressed genes in the former (Figure 1a). This strategy has been successfully carried out by Ruder et al. [28] and Modica et al. [29] to identify putative toxins from the posterior salivary glands of some cephalopods and of the vampire snail *Colubraria reticulata*, respectively. Concomitantly, the analysis of *Eulalia*’s proboscis and body wall whole-transcriptome yielded a total of ≈400,000 contigs, corresponding to ≈55,000 single-gene transcripts in an open reading frame (ORF), as illustrated in Figure 1b. Of these, 2048 were significantly differentially expressed between the proboscis and body wall (adjusted *p* < 0.05), with 718 ORFs showing proboscis-enriched expression (Figure 1c). Translated sequence matching using BLASTp by contrasting against the customized Toxins database retrieved from Uniprot (based on proteins flagged with “toxins” as the functional search term) resulted in 120 amino acid sequences considered to be the most significantly matched against toxin-related proteins, i.e., with the lowest *e*-value (see Table S1), and which are produced in the proboscis of the worm (Figure 1b–d). Altogether, the proboscis and body wall of the worm yielded very distinct transcriptional signatures, from which the 120 ORFs corresponded to proboscis-specific and toxin-related proteins (Figure 1d).

### 2.2. Conserved Domains in Toxin-Like Proteins Reveal the Common Signature of Venoms and Poisons

**Figure 2:**
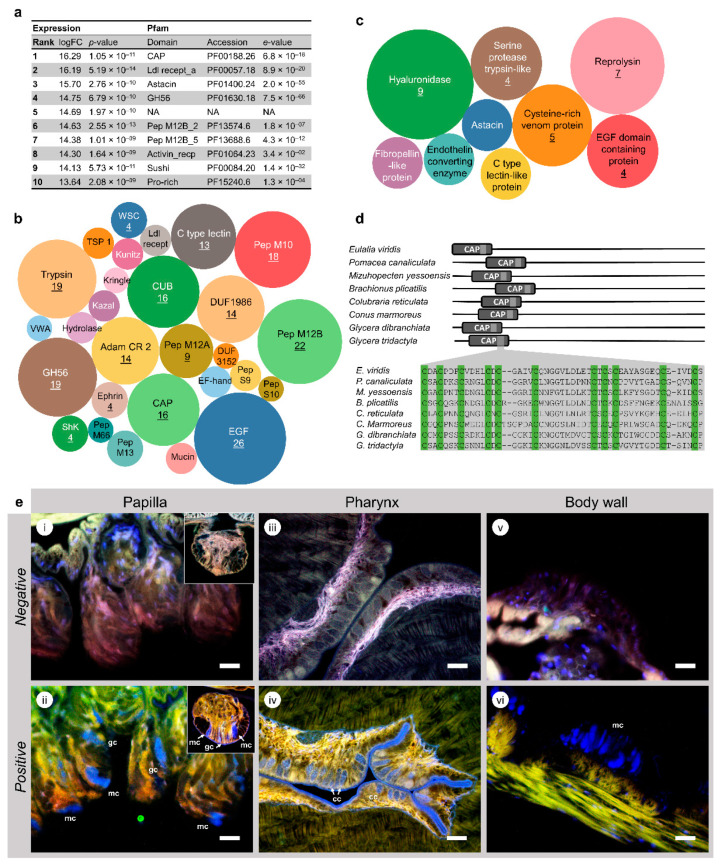
Common signatures of *Eulalia*’s toxins. (**a**) Top ten hits of proteinaceous substances upregulated in the proboscis, compared to the body wall and matched against Pfam. (**b**) Main conserved domains present in proteins with hits in the Toxins database that are overexpressed in the worm’s proboscis (logFC > 2). Figures indicate number of proteins (when ≥4) bearing each domain and the size of circles is representative of number of ORFs. (**c**) Proteins of interest upregulated in *Eulalia* proboscis with logFCs > 10 with match in the Toxins database. Figures indicate the number of different ORFs pertaining to each protein (when ≥ 4). The size of circles represents relative expression. (**d**) Illustrative representation of a cysteine rich venom protein (CRISP) from *Eulalia* sp. and other organisms, highlighting the location of the well-conserved CAP domain and their cysteine alignments. (**e**) Localization of toxin-secreting glandular cell in *Eulalia* through detection of CRISPs by histochemical fluorescent staining of thiols. (i, ii) Negative and positive reactions of thiols, respectively, in the sensorial papillae lining in the outer integument of the proboscis. The reactions produce a blueish fluorescent probe with a positive signal in granular cells (gc) and mucocytes (mc). The morphology of the latter is distinctive due to the presence of large mucus sacculi with homogenous positive staining for thiols due to the presence of sulphated mucins. Specimen fixated with glutaraldehyde. Inset: Detail of individual papillae. (iii, iv) Negative and positive staining in the internal epithelium of the proboscis (pharyngeal epithelium) revealing the single layer of calix cells (cc), which were identified as toxin-secreting cells in our previous work [19]. The granules in these cells yield strong blue fluorescence. Specimen fixated in Bouin’s solution. (v, vi) Fluorescent labeling (negative and positive, respectively) of thiols in the skin was circumscribed to mucocytes (mc). No granular or calix cells were here detected. Specimen fixated in glutaraldehyde. Scale bars: 25 µm.

Among the 718 ORFs with higher expression in the proboscis, those with the highest expression were predominantly toxins (Figure 2a). Analysis then focused on the shortlisted sequences (120 ORFs) with hits in the Toxins database, where a total of 29 types of conserved domains were found (Figure 2b). Several of these domains were represented in more than eight different putative proteins. This is the case of the conserved domains: glycoside hydrolase (GH56), peptidases M12A, M12B and M10, Adam CR 2, C-type lectin, CAP, trypsin, epidermal growth factor (EGF)-like, DUF1986 and CUB (for complement C1r/C1s, Uegf, Bmp1). Several domains are characteristic of peptidases that are highly expressed in the proboscis of *Eulalia*, namely peptidase M13, astacin and reprolysin (the latter two corresponding to peptidases M12A and M12B, respectively), which are zinc-dependent metallopeptidases. These metallopeptidases were also found in the venom of the Polychaeta *Glycera*, believed to be responsible for the digestion of the extracellular matrix of prey, likely acting as permeabilizing agents [2]. In turn, Adam CR 2 is a cysteine-rich ≈ 70 amino acid-long domain, normally paired with the reprolysin domain, both characteristic of metallopeptidase M12B. On the other hand, proteins with EGF-like domains (which are well-conserved domains in Animalia) are ubiquitous among the Eumetazoa and hold several functions [30]. Proteins with this domain are common in venomous cocktails, including astacin and C-type lectin-like. One group of enzymes with conserved domains are hyaluronidases, which are characterized by possessing the long glyco hydro 56-like domains (≈300 amino acid residues). The relatively high number of hyaluronidases’ ORFs (nine sequences) can be, at least in part, better explained by intraspecific variability (e.g., allelic variation or differences in mRNA splicing) leading to multiple transcript variants than by the existence of different genes. However, hyaluronidases, the main function of which is the degradation of glycosaminoglycans such as hyaluronan and chondroitin, are therefore important assets in the digestion and permeabilization of connective tissue. They are particularly well-described in scorpion and bee venoms [31]. In *Eulalia*, a domain of unknown function (DUF), namely DUF 1986 (as identified in Pfam), was one of the most frequent domains in putative toxin-like proteins. This yet uncharacterized 114-amino acid residue domain has been found in proteins of the trypsin-like serine protease superfamily, which is well-represented in venomous cocktails, as well as for other serine proteases. These proteases can also have CUB domains (100 amino acids) and are well-described in honeybee venoms, for instance [32]. These domains (referred to as CUB1 and 2), although ubiquitous and associated with multiple functions, are seemingly restricted to extracellular or transmembrane proteins and are primarily involved in the mediation of protein–protein interactions, including the enhancement of proteolytic activity directed against the extracellular matrix, such as in the case of procollagen proteinases [33,34]. Overall, the most important conserved domains in toxin-related proteins secreted by the proboscis of *Eulalia* are cysteine-rich proteins and enzymes capable of extensive activity against the extracellular matrix, which have a primary role long acknowledged for enzymatic toxins in venomous cocktails. The composition of the mixture of proteins in *Eulalia* holds, in fact, higher similarities to *Glycera* than crustaceans [1,2].

### 2.3. Cysteine-Rich Neurotoxins Are a Major Component of Eulalia’s Toxungen

Thirty-eight ORFs with significant representation in the Toxins database were found to be highly proboscis-specific, inferred from high expression ratios (logFC > 10). These ORFs represent multiple transcripts for nine different proteins (Figure 2c). In terms of representativeness, hyaluronidase leads the subset with nine sequences, followed by reprolysin with seven and then cysteine rich venom protein (CRISP) with four. Indeed, cysteine-rich venom CRISP-like proteins were well-represented in this subset, but they markedly contrasted with the majority of the proteins, which consisted of enzymes acting on the extracellular matrix, therefore involved in tissue permeabilization. Furthermore, one of the CRISP ORFs was found to be the most significantly upregulated among all matches (see Figure 2a), attaining high expression ratios (logFC > 16) between the proboscis and the body wall.

Cysteine-rich proteins holding CAP domains are known to be key components in animal venoms, from terrestrial, such as snakes, to marine, including the relatively well-studied cephalopods [7,28]. In *Eulalia* and other species from various taxa, CRISPs have a well-preserved CAP domain at the N-terminus and several cysteine residues throughout the amino acid sequence (exemplified in Figure 2d). These glycoproteins, which in mammals are secreted in the epididymis (albeit their function remaining unclear), have been persistently found in venoms, being well-described in snakes. Mostly, they act as neuromuscular toxins by blocking calcium channels, but they can also function as potassium channel blockers to prevent muscle contraction [29,35].

Data indicate that CRISP-like proteins are the neurotoxic agents secreted by the proboscis of *Eulalia*. Indeed, these findings are in accordance with our previous work with *Eulalia*, in which they were named phyllotoxins and their neuromuscular effects were first characterized by means of bioassays and observation in the natural habitat with some of the worm’s favorite prey, namely mussels and other Polychaeta [15]. We have previously shown that *Eulalia*, being devoid of jaws, stuns its prey by repeated contact with specialized tentacles at the tip of the proboscis (see Cuevas et al. [15] for videographic evidence) and at the base of which (lining the interior of the proboscis) are located calix cells likely responsible for the secretion of putative neurotoxins delivered by mucus [19]. These morphological and behavioral findings, together with transcriptomics, are in agreement with requisites for the identification of venomous organisms [3].

Since one of the main characteristics of cysteine-rich domains is the presence of a highly reactive thiol side chain, we then hypothesized that CRISPs could be localized histochemically using a protocol for fluorescence microscopy. This protocol provided the confirmation of gland cells location and provided phenotypic anchoring for CRISP differentially expressed genes (DEGs). The findings (summarized in Figure 2e) revealed the existence of thiol-rich granular cells in the sensorial papillae that cover the fibrous integument of the proboscis and in the calix cells within the epithelium that lines the inside of the proboscis. These two types of cells are not present in the main body wall. There are, however, mucocytes that stain positively for thiols, likely due to sulphated mucins. The cellular structure of mucocytes and granular or calix cells has already been comprehensively described elsewhere [19]. Here, the homogenous appearance of the large mucus sacculi clearly contrasts with the dense, much smaller and regular-shaped protein granules that characterize calix cells. Mucocytes are, nonetheless, widespread throughout the surface of the animal, unlike granular cells in the proboscis. The latter are then certainly responsible for the differential expression of CRISPs between the two organs, further supporting the proboscis as toxin-secreting (and delivery) structure.

Besides CRISPs, metalloendopeptidases and hyaluronidases were the most representative proteins matching with those in the Toxins database and within the group of highly overexpressed protein homologs in the proboscis (Table S3). Despite the reduced number of works on Polychaeta, these enzymes have already been reported to be well-represented in venomous secretions of *Glycera* and the Amphinomidae fireworms, with the exception of hyaluronidases and trypsin-like serine proteases [2,14]. Nonetheless, even the latter two are listed as being typically present in venoms from gastropods, cephalopods and snakes [28,29,36]. In snakes, hyaluronidases are reported to contribute to widespread inflammation and enhanced venom diffusion [37]. Endothelin-converting enzymes, another group of metalloproteases, were also found to be significantly overexpressed in the proboscis of *Eulalia*, similarly to the venomous cocktails of various animals, like scorpions and snakes [38,39]. As they degrade amyloid beta (the accumulation of which is linked to Alzheimer’s disease), it is yet another compelling factor encouraging the study of venom components for biomedical applications [39].

Altogether, enzymes targeting the extracellular matrix, combined with the membrane-disrupting and toxin dissemination roles of hyaluronidases described in snakes, bees and even in the intestine of humans, as facilitators of absorption [31,40,41], assist in the diffusion of *Eulalia*’s cysteine-rich neurotoxin, phyllotoxin. In addition, the anticoagulant activity of C-type lectins [42] and the pro-hemorrhagic activity of metalloproteinases from the family M12 [36,43] hinder healing, facilitate infiltration of toxins and favor the extraction of fresh pieces of tissue via suction while the prey is chemically stunned.

### 2.4. Phylogenetic Analysis of Individual Components

**Figure 3:**
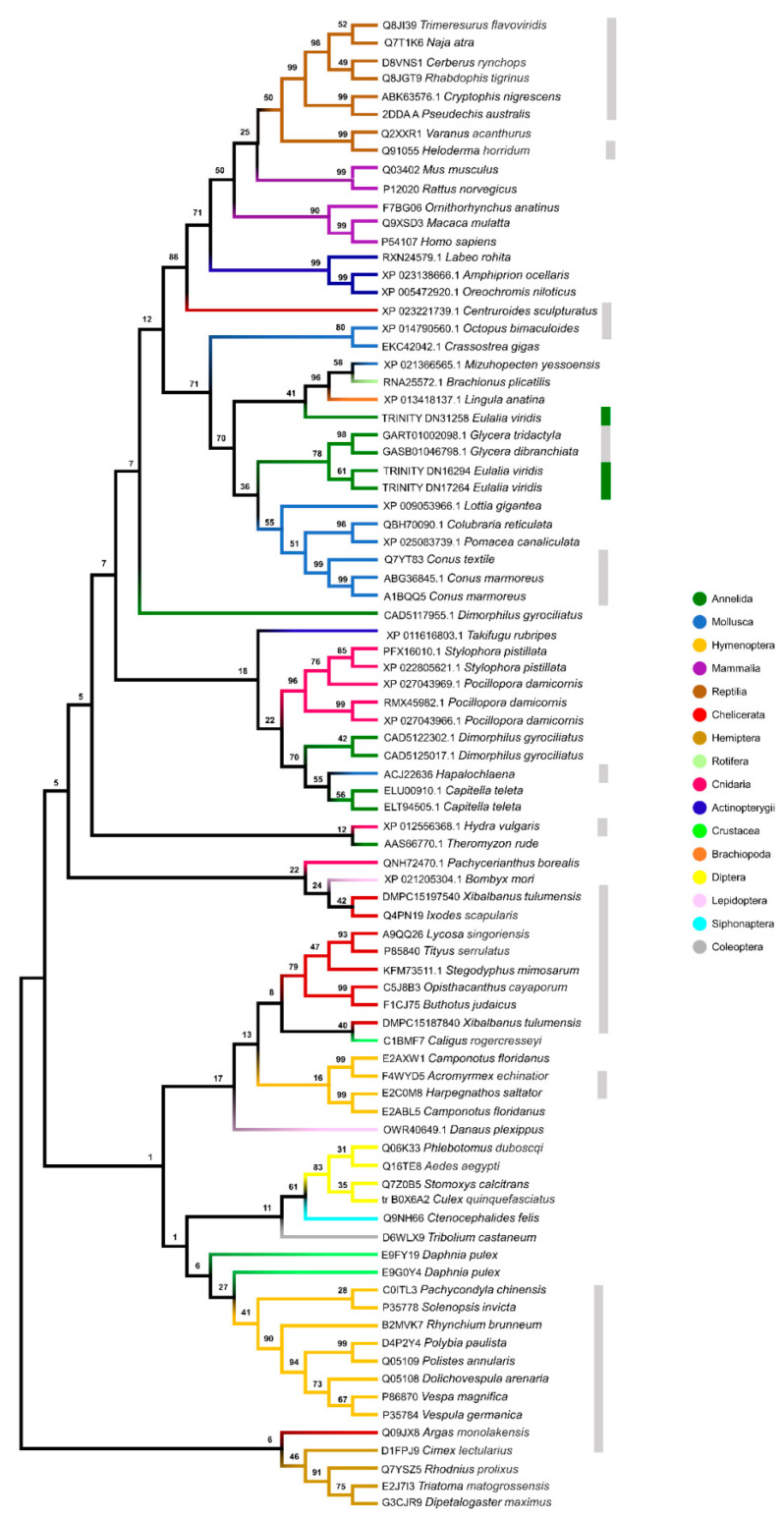
Phylogenetic trees of CRISPs secreted by *Eulalia*. The model includes the most overexpressed CRISP sequences in the worm and the best matches from venomous and non-venomous animals for comparison. The phylogenetic reconstruction was made with MEGA X using the Whelan and Goldman model with discrete gamma distribution, with 1000 bootstrap pseudoreplicates. Bootstrap support values are given for all nodes and clade names are indicated by colored branches. Grey bars indicate known venomous or toxins-bearing organisms and the green bars indicate *Eulalia* homologs.

**Figure 4:**
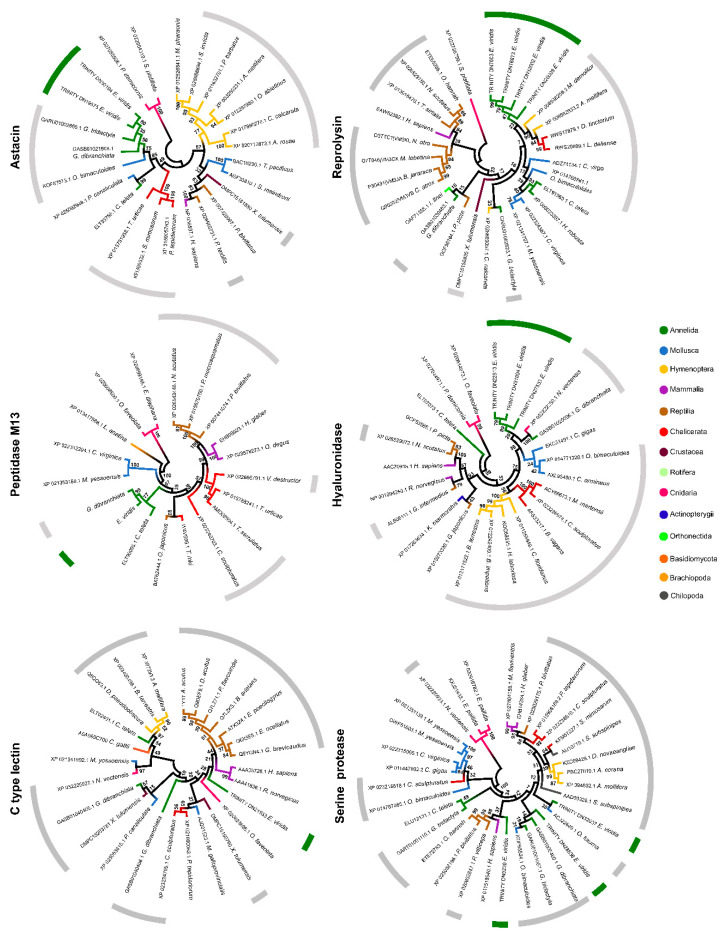
Phylogenetic trees of some of the major toxins secreted by *Eulalia*. The models include the most representative sequences in the worm and the best matches from venomous and non-venomous animals for comparison. The phylogenetic reconstruction was made with MEGA X, with 1000 bootstrap pseudoreplicates. Bootstrap support values are given for all nodes and clade names are indicated by colored branches. Grey bars indicate known venomous or toxins-bearing organisms and the green line indicates *Eulalia* homologs.

The sequences of shortlisted proteins (recall Figure 2c) were matched against their homologs with the most significant hits amongst venomous and non-venomous representative eumetazoans, from cnidarians to humans, plus against other organisms that produced relevant matches, like Basiodiomycota (Fungi) and Orthonectida mesozoans, which, when available for comparison, were analyzed based on phylogenetic models (Figure 3, Figure 4 and Figure S2). Interestingly, the trees show different phylogenetic relationships for each protein, albeit with a trend of grouping the proteins from *Eulalia* within clades that allocate homologs from venomous animals, especially *Glycera*, a known venomous Phyllodocida. Although most *Eulalia* homologs are closely related with *Glycera*, most clades organization does not clearly reflect the known evolutionary history of eumetazoans. If, on the one hand, the findings confirm the positioning of *Eulalia* protein homologs within common constituents of venomous secretions, on the other hand they evidence that the evolution of the mixture of the substances that take part in a venom system is complex. Indeed, it has been argued that no single evolutionary model can explain how toxins evolved from non-toxin substances [9]. At the molecular level, phenomena such as mutations, gene duplications and differential regulation of gene expression and mRNA maturation are likely involved. However, it is the ecology of each species and, therefore, the evolutionary pressure set upon it that ultimately dictate the turn of proteins into toxins, permeabilizing agents and other accompanying substances common in venomous secretions. Jackson and Koludarov [9] even proposed that a distinction be made between two terms commonly used by toxinologists: “neofunctionalization”, which stands for the acquisition of a novel function by any means, and “recruitment”, which implies that the existence of molecule within a venomous cocktail turns it into a toxin for a specific purpose. It is plausible that in *Eulalia*, as in other eumetazoans, normal proteins were “recruited” to function as toxins or adjuvants (such as metallopeptidases), whereas more specific substances, such as cysteine-rich neurotoxins, were neofunctionalized into toxins. The specific molecular processes underlying these changes remain at this stage conjectural, especially in light of what is not yet known about the genomes of Polychaeta, their evolutionary history and diversity of venom systems.

It should be highlighted that the CRISPs (Figure 3) from *Eulalia* were phylogenetically closer to those of *Glycera*, arguably one of the best-known venomous Polychaeta, and mollusks, including the genus *Conus*. The other non-venomous Annelida included in the model were not closely related with *Eulalia* homologs. As such, a common origin in cysteine-rich neurotoxins may be suspected among the Phyllodocidae Polychaeta. von Reumont et al. [2] already discussed the presence of cysteine motif-bearing neurotoxins in *Glycera* and their potential similarity to cnidarian gigantoxins. Gigantoxins are, in their turn, members of the epidermal growth factor family and their neurotoxic activity has been described in various invertebrates, like *Pomacea* (Gastropoda) and sea anemones [44], which explains the resemblance of *Eulalia* EGF domain-containing proteins to a broad clade of organisms, spanning from *Platynereis*, another Phyllodocida, to humans and even rotifers (see Figure S2). Altogether, these data indicate that phyllotoxins may, in fact, refer to a family of multiple cysteine-rich neurotoxins that are ubiquitous among Phyllodocida, at least, and result from an ancient radiation.

Another significant case is that of hyaluronidases, which are ubiquitous amongst animal venoms and well-conserved between eumetazoans, venomous or not, as *Eulalia* homologs are similar to those of *Glycera* and a venomous Cnidaria, as well as closely related with mollusks. These enzymes, combined with zinc peptidases, namely serine proteases, astacins, reprolysins and peptidase M13, show the incorporation of diverse enzymes directed against the extracellular matrix of prey. The peptidase M13 and astacin show the most expected homology, with higher similarities with annelids and mollusks. Reprolysin, in turn, is more closely related with venomous Chelicerata and Hymenoptera than with annelids and mollusks. Serine protease was the only protein with very distinct isoforms. Even with the major clades belonging mainly to Mollusca and Annelida, the isoforms are related to different proteins. One isoform is closely related to a serine protease from *Homo sapiens* and reptiles, another with an S1 type peptidase from venomous octopuses and the latest with a serine protease from *Glycera*. Even though all the sequences have the trypsin-like serine protease conserved domain, the presence of CUB domains in some variants may indicate that some sequences are toxins whereas others may be involved in development. It must be noted, however, that reduced annotation may challenge the functional characterization of these proteins among taxa.

In turn, C-type lectin from *Eulalia* formed a distinct group with reptiles and mammals, with *Glycera* being positioned farther away. It should be noted, however, that the C-type lectin-like sequences retrieved for snakes consist of calcium-dependent anticoagulant factors, inhibiting both intrinsic and extrinsic coagulation pathways [45]. These proteins, now believed to be major components of snake venoms, are also known to hinder coagulation and promote the lysis of blood cells [46,47,48]. Interestingly, a protein similar to C-type lectin was found in *Cryptococcus gattii* (Fungi: Basidiomycota), a tropical pathogenic yeast that requires interaction with the immune system of hosts, macrophages specifically [49]. This sequence was clustered together with bees (curiously with the innocuous model marine Polychaeta *Capitella teleta* as well), which indicates recruitment of these proteins for distinct roles within venoms according to the ecology of the species, such as anti-immune and anti-coagulant roles. Altogether, these data indicate that, rather than evolving as a whole, these proteins converged to form a cocktail with three major functions: immobilizing, permeabilization and tissue disruption plus impairment of coagulation. These properties facilitate the extraction, making use of the powerful pharyngeal musculature, of fresh tissue from marine invertebrate prey that do not possess competent immune systems to clear toxins but that are still provided with efficient healing, at least for Polychaeta [50].

### 2.5. Multigene Phylogeny

In the face of the inconsistent phylogeny of individual components of *Eulalia*’s toxungen, we hypothesized that the composition of the mixture as a whole could provide insights not only into how the mixture evolved in *Eulalia* but also how venoms may have functionally adapted among marine animals. After retrieving homologs for the eight shortlisted toxins in *Eulalia* (astacin, reprolysin, CRISP, hyaluronidase, C-type lectin, serine protease, EGF domain-containing protein and endothelin-converting enzyme), eighteen species pertaining to eight major Eumetazoan groups, from Cnidaria to Mammalia (the latter as non-toxin homologs) were included in the multitrait model (Table S4). The species were chosen according to the availability of sequences and broad representativity. Cnidarians (two species) were included due to their lower positioning in the animal tree-of-life. The presence of all eight proteins in both cnidarian species is an indication that these proteins derive from ancient radiations [51]. Indeed, the full phylogenetic model (Figure 4) shows Cnidaria, represented here by the anemone *Exaiptasia pallida* and by the coral *Pocillopora damicornis* (both considered innocuous), forming a clade clearly distinct from all other groups. The Polychaeta, represented by *Eulalia* and *Glycera* (toxin-secreting) plus the non-harmful *Capitella* (a small annelid whose interest as a model organism is growing), and the scallop *Mizuhopecten* form a distinct group from that which irradiated into two clades, one of which holds a clade that allocates all vertebrate species in the tree, venomous or not. The close association between *Eulalia* and *Glycera* is no surprise, as they are both predators belonging to Phyllodocida. Their respective families (Phyllodocidae and Glyceridae) are regarded as sister groups within the Annelida [24,25]. These two groups, together with known venomous Polychaeta, namely fireworms (Amphinomidae) [14], point towards the possibility that the Phyllodocida harbor more toxin-secreting species than was perhaps anticipated. The phylogeny of these proteins in invertebrates, protostomes in this case, namely Arthropoda and Mollusca, is, however, more elusive. In fact, arachnids and mollusks are dispersed by two distinct clades that do not reflect the known tree-of-life. Nonetheless, among these two groups, only the snail *Pomacea canaliculata* is considered harmless.

The tree indicates that, among protostomes, venoms of Polychaeta derive from a more ancient radiation than anticipated. Furthermore, the acknowledged phylogenetic proximity between Annelida and Mollusca is not seen in the model, with the exception of the positioning of the scallop (a non-venomous organism), which are the most important groups of the Lophotrochozoa, based on both molecular and morphological data [52]. These findings indicate that toxin-bearing secretions from *Eulalia* and, possibly Polychaeta in general, became functionally adapted independently of the eumetazoan life history. On the other hand, prominent molluscan toxins, like conotoxins (which are potent neurotoxins), have not only been found to have evolved relatively recently [51] but have also been found to have a relatively distant association to the sequences retrieved from *Eulalia*, especially CRISP and EGF domain-bearing neurotoxins (albeit within the same wide clade), which indicates convergent adaptation against neuromuscular activity. Indeed, the venom of *Conus* is nowadays considered to be a very refined specialization, at least with respect to potency and specificity, to which can be added the relatively reduced molecular weight of conotoxins. These are typically comprised of 10–20 amino acid residues, which can be compared, for instance, with snake CRISPs, which have about 100 [51,53], and *Eulalia* CRISP-like proteins, with at least twice as many. These differences did not hinder clustering *Eulalia* and *Conus* CRISPs within the same major clade, as shown in Figure 3, which indicates a common ancestor. In turn, cephalopod and arthropod venom proteins have arisen in distinct moments of evolution after irradiation from non-toxin homologs, potentially originating toxin and non-toxin paralogs if duplications occurred. In fact, the importance of the retention of gene duplicates that code for toxins in the evolution of venoms has already been noted (see the review by Wong and Belov [54]), even though the subject represents little more than uncharted ground with respect to marine invertebrates. Still, the resemblance of *Eulalia* toxins to non-toxin homologs (as shown in Figure 3 and Figure 4), as well as *Eulalia*’s positioning with regard to *Capitella* and *Mizuhopecten* in the multitrait model, further validates this premise. Other authors have nonetheless pointed out that post-transcriptional mechanisms, such as alternative splicing, or even post-translational cleavage can be responsible for the expression of a wide variety of toxins, which makes it difficult in any case to identify orthologues and evaluate orthologous expression of toxins [54]. It should be highlighted that similar reasoning can be applied to vertebrates, which appeared clustered in a single clade in the multitrait model, combining venomous and non-venomous animals in a monophyletic branch that, in this case, closely mirrors the expected tree-of-life. It is likely that the growing interest in marine invertebrate toxins and the rapid advances in next-generation sequencing methods will bring about important findings in the near future as new species of venomous animals are unraveled. It should be highlighted that the phylum Annelida is probably not monophyletic, with the recent addition of the previously considered individual phyla Sipuncula and Echiura [55] showing that much of the taxon’s phylogeny and systematics remain unresolved. Additional challenges are provided by the lack of genomic annotation, despite the promises of *Capitella*, and considerable intraphylum genomic variability [56]. Associated with increased taxon sampling as a means to tackle phylogenetic uncertainties [57], venomics can thus provide important clues to the origins of venom proteins, their function and their relation to the animal’s milieu, either as part of its mechanisms of predation or as defense against predators [58].

**Figure 5:**
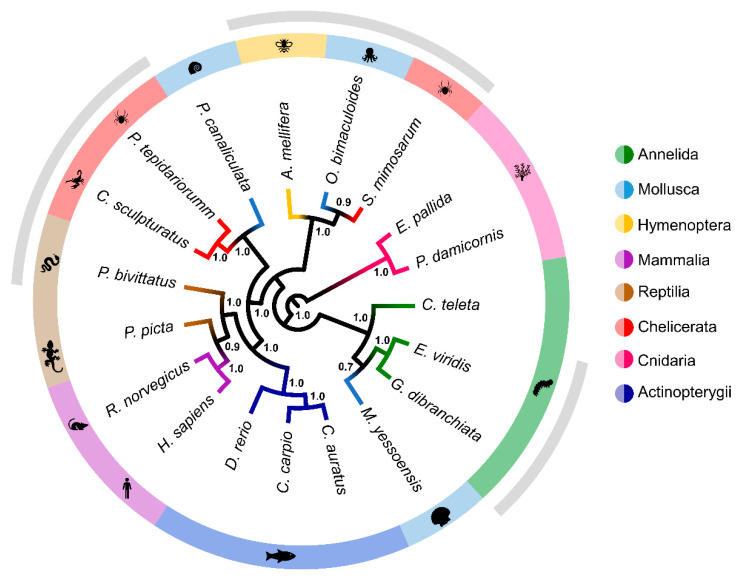
Multigene phylogenetic tree combining major toxin homologs: CRISP, hyal, astacin, reprolysin, C-type lectins, serine proteases, endothelin-like enzymes and EGF domain-containing neurotoxins. The tree was produced using Bayesian inference (1,000,000 generations, samples recorded every 100 generations) with MrBayes 3 (see Table S4 for sequence information). Clade credibility values are given for all nodes and taxonomic groups names are indicated by colored branches. Grey bars indicate known venomous organisms.

Sunagar and Moran [51] proposed the “two-speed” theory of venom evolution, according to which more ancient animals invested in the diversification of toxins as means to assure a broad range of prey whereas “younger” species invested in specialization, potency and reduced energetic costs. Within this perspective, the multitrait model given in Figure 5, plus the wide scope of putative, high-molecular weight toxins from *Eulalia* (many of which are not yet characterized) and the mild potency of its neurotoxins in mussels [15], places this organism in the first group. Younger taxa, such as *Conus*, for instance, more likely pertain to the second. Given the ancient radiation of Polychaeta and the wide span of prey of *Eulalia*, it is thus reasonable to assume that the species, and likely other Phyllodocida, responded to a positive selective pressure to diversify their toxin arsenal.

## 3. Conclusions

The evolution of animal venom systems is complex and may involve multiple episodes of protein recruitment and neofunctionalization from non-toxic proteins. In Polychaeta, especially phyllodocids, venom systems can be common features that have evolved to favor feeding diversity, albeit compromising potency, specificity of target and metabolic costs. Following multiple lines of evidence, from sequence homology matching, searching for conserved domains and phylogenetics to ecology and toxicology, *Eulalia* now joins the ranks of venomous (or, better, toxungen-bearing) marine organisms. In *Eulalia*, the toxins have three major roles: (i) hindering the neuromuscular activity of prey; (ii) pro-hemorrhagic and anti-clotting, and (iii) permeabilization and liquefaction of tissue as a mean to assist neurotoxin diffusion and assist feeding through suction. Indeed, proteinaceous animal venoms hold a common signature, with particular emphasis on blockers of neuromuscular activity and agents that assist their infiltration. The evolution of individual toxins is thus not straightforward, as proteins have likely been recruited at different stages of the species’ life history and by various processes at the molecular level. In the near future, we may expect an increase in the relevance of chemical warfare in marine animals for the understanding of eumetazoan phylogeny, in line with the growing biotechnological interest in toxins and other bioreactives from the organisms that have evolved to adapt to the world’s most vast and diversified ecosystems.

## 4. Materials and Methods

### 4.1. Animal Collection

*Eulalia* sp. pertaining to the complex *E. viridis*/*E. clavigera* (≈120 mm total length and weighting ≈ 250 mg) were collected from a rocky intertidal beach in Western Portugal (38°41′42″ N; 09°21′36″ W). Animals were kept in a mesocosm environment recreating their natural habitat, consisting of dark-walled glass aquaria equipped with constant aeration and recirculation, fitted with natural rocks and clumps of mussels to provide shelter and feed, as set-up by Rodrigo et al. [59]. Salinity, temperature and photoperiod were kept within 35, 16 °C and 12:12 h, respectively.

### 4.2. RNA Extraction and High-Throughput Sequencing (RNA-seq)

To identify mRNAs coding for putative proteins in the proboscis, which is involved in toxin secretion and delivery, the body wall (which includes skin and underlining musculature) was taken as reference organ. Worms were dissected for the excision of proboscis and body wall. Extraction of total RNA was done on portions infiltrated with RNALater using the RNeasy Protect Mini Kit coupled with in-column DNA digestion using an RNAase-free DNAase set (all from Qiagen, Hilden, Germany), following manufacturer instructions. Quantification of total RNA and initial quality assessment was performed using a Nanodrop 1000 spectrophotometer (Thermo Fisher Scientific, Waltham, MA, USA). Samples were stored at −80 °C until further analysis. The RNA integrity number (RIN) for each sample was determined in an Agilent 2100 Bioanalyzer (Agilent Technologies, Santa Clara, CA, USA), with all samples being found to be within suitable parameters, i.e., intact or partially degraded samples with RIN ≥ 7, input of ≥1 μg total RNA, free of contaminating DNA [60]. Library preparation was done using Kapa Stranded mRNA Library Preparation Kit and the generated RNA fragments were sequenced in an Illumina HiSeq 4000 platform, using 150 bp paired-end reads. A sample from the proboscis (pr) and another from the body wall (bw) were sequenced with high depth for transcriptome assembly (100 M reads) and normal coverage (20M reads) was employed in two further biological replicates.

### 4.3. Transcriptome Data Analysis

The quality of RNA-seq data was assessed using FASTQC (v0.11.7, https://www.bioinformatics.babraham.ac.uk/projects/fastqc/). For all libraries, TrimGalore (v0.4.4, https://www.bioinformatics.babraham.ac.uk/projects/trim_galore/) was used to: trim the 13 bp of the Illumina standard adapter; remove low quality reads with a “Phred” cut-off lower than 20; and set the minimum read length to 20 bp. An average of 10% of low-quality reads were removed from each sample. Quality filtered reads from high-depth sequenced samples were combined and assembled with Trinity v2.8.4 [61] using default parameters. Two strategies were used to evaluate the quality of the assembled transcriptomes: examining the RNA-seq read representation of the assembly (at least 80% of the reads); and computing the Ex90N50 transcript contig length (approximately 1000 bp). Coding regions within the assembled transcriptomes were predicted using TransDecoder v5.5.0 [62], with default parameters. Proboscis-specific transcripts were selected by first independently mapping all samples to the *Eulalia* assembled transcriptome with Kallisto v0.44.0 [63] and then selecting the transcripts significantly overexpressed relative to the body wall (FDR-adjusted *p* < 0.05), with expression fold-change higher than two. Statistical analyses were computed using R 3.5 [64], through the packages edgeR and limma. Finally, proboscis-specific transcripts with coding regions were functionally annotated by scanning for homology against: (1) UNIPROT cluster UniRef90 [65] by generating a customized database based on proteins flagged with “toxins” as the functional search term in BLASTP v2.5.0 [66], having set a maximum *e*-value of 10^−5^; and (2) protein domains from PFam [67], using HMMER v3.1b2 [68]. Bulk data is freely accessible at Gene Expression Omnibus (GEO) DataSets database, accession number GSE143954.

### 4.4. Quality Assessment and Validation

The results from RNA-seq were validated by reverse transcription quantitative polymerase chain reaction (RT-qPCR) for the selected representative genes as described in Rodrigo et al. [69]. In brief: cDNA was synthetized from the total RNA samples (obtained as described in Section 4.2) using the First-Strand cDNA Synthesis Kit (NZYTech, Lisbon, Portugal). Primers were designed using Primer Blast and verified in silico with Oligo Analyzer (Table S2) to amplify an expressed sequence tag (EST) for the selected genes (Table S3), namely hyaluronidase, cysteine rich venom protein, C-type lectin-like protein and metalloproteinases M12A and M12B, and GAPDH as internal control, as suggested by Thiel et al. [70]. Amplification was performed in a Biometra Gradient Thermocycler96 (Analytik Jena, Jena, Germany). Following resolving PCR products in an agarose gel, these were Sanger sequenced, translated and matched against the Toxins database using BLASTP. All products were found to align with the desired targets. The RT-qPCR was then performed in a Corbett Rotor-Gene 6000 thermal cycler (QIAGEN, Hilden, Germany) using the NZY qPCR Green Master Mix (NZYTech). The program included an initial denaturation (95 °C, 10 min), followed by 45 cycles of denaturation (94 °C, 45 s), annealing (54 °C, 35 s) and extension (72 °C, 30 s). Expression analysis was done using the ∆∆Ct method [71] (Figure S1). Primer melting analysis was also conducted to verify specificity of hybridization.

### 4.5. Multigene Phylogenetics

To compare the sequences of toxungenous components between several clades, we first produced a shortlist of translated contigs with logarithmic fold changes (logFCs) above 10 from the top hits extracted from the customized toxins database and Pfam. A total of 38 genes were selected, corresponding to nine different proteins (isoforms concatenated). Sequences from each protein were chosen to scan for homology against NCBI’’s RefSeq, and UNIPROT databases using Blast [72] when necessary, with the following clade restrictions: Mammalia, Arachnida, Cephalopoda, Annelida, Hymenoptera, Bivalvia, Reptilia, Serpentes, Scorpionida, *Conus*. The best hits without restriction were also used. After alignment, the best model for each toxungen was selected according to the lowest Bayesian information criterion (BIC): the Whelan and Goldman (WAG) model with discrete gamma distribution (G) and evolutionarily invariable (I) for peptidase M12A, the WAG+G model for CRISP and serine protease, the Le Gascuel model (LG) plus G+I for peptidase M13 and WAG+G+F (frequencies) for EGF domain-containing protein, hyaluronidase, C-type lectin and peptidase M12B. Phylogenetic trees were produced (1000 bootstrap pseudoreplicates) for the sequences of each individual protein using maximum likelihood, following Tamura and Nei [73]. Sequence alignment and trees were produced with Mega X [74].

The multigene phylogenetic analysis was done from amino acid sequences selected from top hits of 18 key species representative of different phyla by homology with hits for the eight genes encoding the shortlisted proteins of interest (CRISP, hyaluronidase, C-type lectin, serine protease, peptidase M12A and M12B, EGF domain-containing protein and endothelin-converting enzyme). Genes were partitioned for analysis and the best model for each was selected according to the lowest Bayesian information criterion: the Whelan and Goldman model with discrete gamma distribution and evolutionarily invariable for peptidase M12B, the WAG+G model for CRISP, C-type lectin, serine protease and endothelin, the Le Gascuel model (LG) plus G+I for peptidase M12A and EGF domain-containing protein and LG+G for hyaluronidase. The aligned and translated sequences are compiled in Table S4. The Bayesian phylogenetic tree was inferred using MrBayes 3 [73] after 1,000,000 generations and sampling every 100 generations. The tree was rooted on the Cnidaria.

### 4.6. Microscopy

Histological samples were prepared for both light microscopy and scanning electron microscopy (SEM). The external structure was analyzed by SEM for morphological characterization, following the protocol described by Inoué [75] and modified by Rodrigo et al. [19]. The internal structure was analyzed by light microscopy for phenotypic anchoring through the localization of cells producing cysteine-rich proteins. Identification of thiol groups histochemically was performed using the Invitrogen Protein Thiol Fluorescent Detection Kit (Thermo Fisher Scientific), as described by Gonçalves and Costa [76]. In brief: slides with samples fixed in glutaraldehyde or Bouin’s solution were embedded in paraffin. The samples were then deparaffinated, rehydrated and stained with detection reagent, after being permeabilized (with 1% *v/v* Triton) and reduced with 1% *m/v* DTT to free oxidized thiols. The procedure was done using a humidity chamber. Negative control slides, without detection reagent, were included for quality assessment. The slides were mounted with DAPI for nuclei staining, photographed using a DM 2500 LED model microscope equipped with a MC 190 HD camera (both from Leica Microsystems, Wetzlar, Germany) and fluorescence images in all channels were treated with ImageJ [77] (Figure S3).

## Data Availability

The data analyzed in this study are openly available at https://www.ncbi.nlm.nih.gov/geo/query/acc.cgi?acc=GSE143954.

## Supplementary Materials

The following are available online at https://www.mdpi.com/2072-6651/13/2/97/s1, Figure S1: Expression analysis of key toxins by RT-qPCR, comparing the proboscis and body wall, Figure S2: Phylogenetic trees of EGF domain-containing protein and endothelin converting enzyme, Figure S3: Channel-split fluorescent-labeled thiols in various tissues of *Eulalia* as markers for CRISPs, Table S1: Hits of proteinaceous substances upregulated in the proboscis, compared to the body wall, with matches against Toxins database, Table S2: Primer sequences, Table S3: Translated amino acid sequences from *Eulalia* used in phylogenetic analyses, Table S4: Accession numbers of sequences employed in multitrait phylogenetics.
