# Supplementary material for: A Transcriptomic Approach to the Recruitment of Venom Proteins in a Marine Annelid
Source: Toxins (Basel). 2021 Jan 28;13(2):97. doi: 10.3390/toxins13020097 (PMC7911839; doi:10.3390/toxins13020097)
Supplement: Supplementary file 1 [file toxins-13-00097-s001.zip › toxins-1051738-supplementary.pdf]

Supplementary Material: A Transcriptomic Approach to the Recruitment of Venom Proteins in a Marine Annelid

Ana P. Rodrigo, Ana R. Grosso, Pedro V. Baptista, Alexandra R. Fernandes and Pedro M. Costa

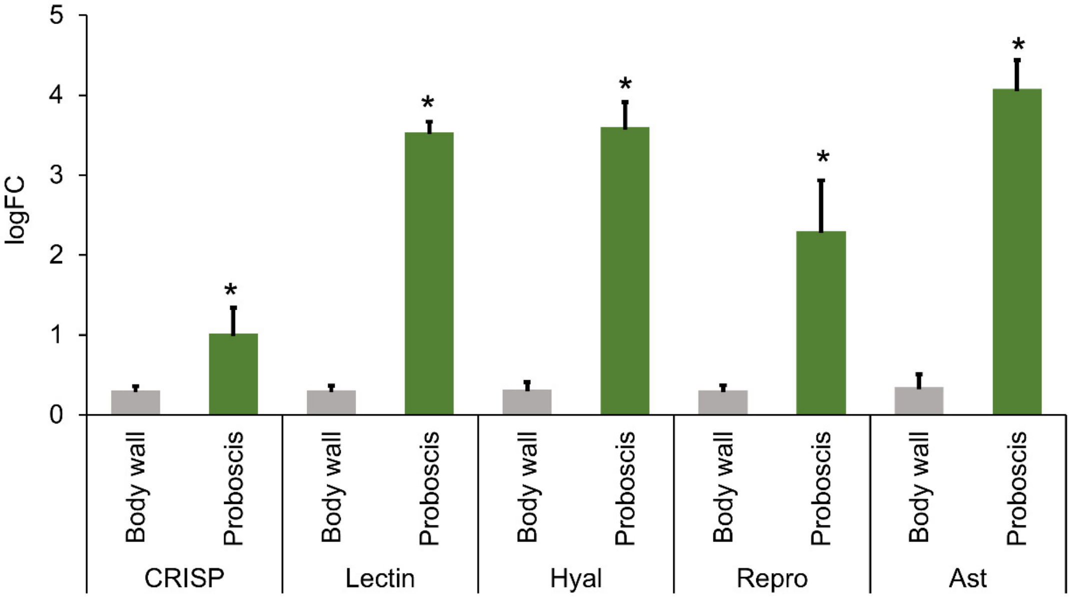

Figure S1. Expression analysis of key toxins by RT-qPCR, comparing the proboscis and body wall. Data are expressed as mean relative expression of *Crisp* (cysteine-rich venom protein), *Lectin* (C type lectin), *Hyal* (hyaluronidase), *Repro* (peptidase M12B) and *Ast* (astacin, peptidase M12A). The housekeeping gene *Gapdh* was used for normalization. * Indicates significant differences to body wall for each respective target (Kruskal-Wallis H, $p < 0.05$).

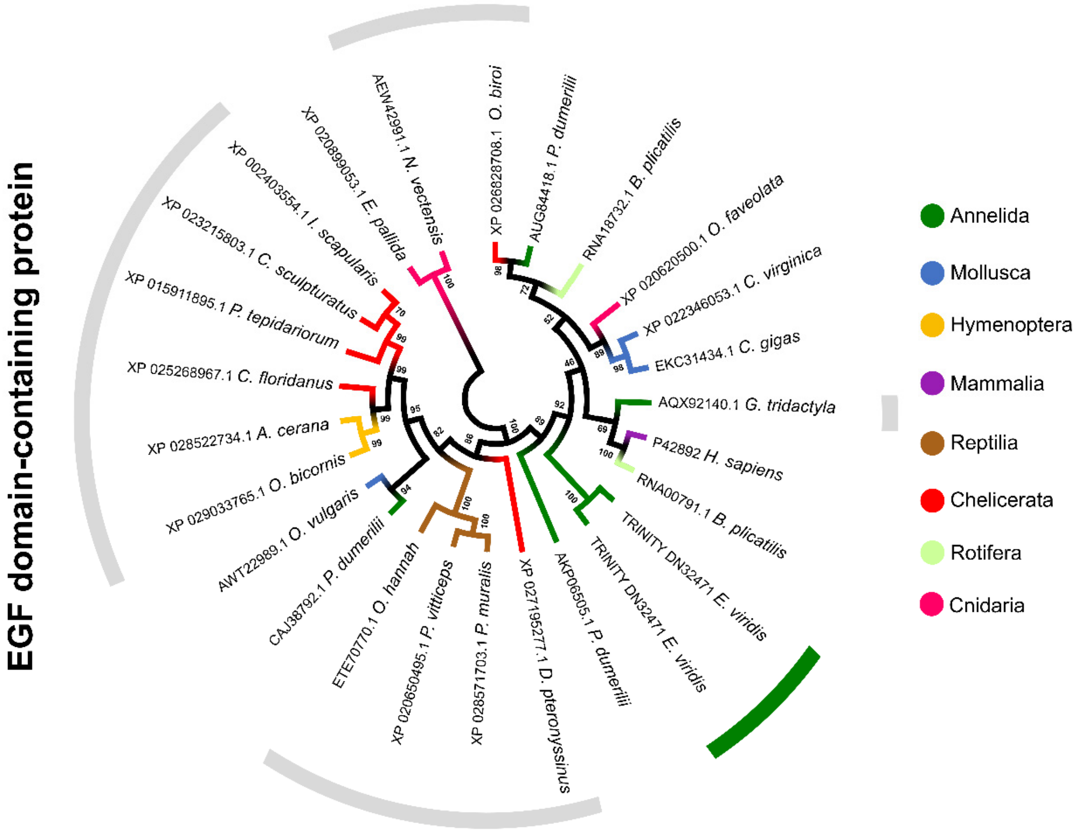

Figure S2. Phylogenetic trees of endothelin converting enzyme. This protein was shortlisted from ORFs with higher relative expression in the proboscis and best-matched against the Toxins database. Phylogenetic reconstruction was made with MEGA X, with 1000 bootstrap pseudoreplicates. Bootstrap support values are given for all nodes and clade names are indicated by colored branches. Grey bars identify known venomous or toxins-bearing organisms, whereas the green bar identifies *Eulalia* homologs.

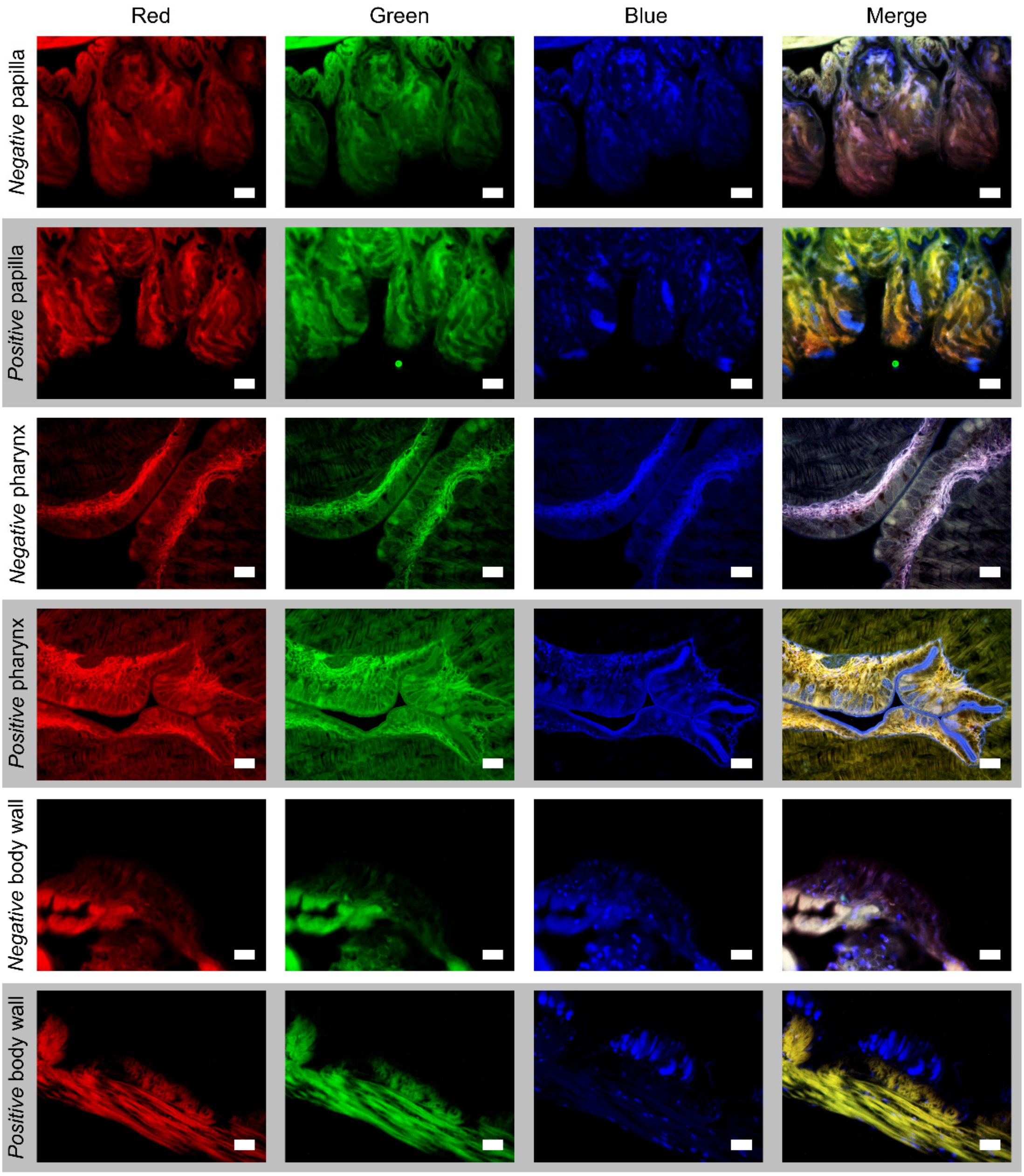

Figure S3. Channel-split fluorescent-labelled of thiols in various tissues of *Eulalia* as a marker for CRISPs. The histological (paraffin) sections were stained with a fluorescent histochemical marker for thiols after treatment with a reducing agent (DTT), producing a bluish labelling. Nuclear counterstaining was produced with DAPI. Negative controls were obtained by treating with DTT but no marker. Here are shown the Individual RGB channels plus respective composite image. Papilla and body wall samples were fixated in glutaraldehyde and pharynx samples in Bouin's solution. Papilla and pharynx present in the proboscis stained positive for thiol groups (granular blue cells) and mucocytes (due to the presence of sulphated mucins). Conversely, sections from the sensorial papillae and body wall stained positive only for mucocytes, which explains the differential expression of CRISPs (higher in the proboscis). Scale bars: 25 µm.

Table S1. Hits of proteinaceous substances upregulated in the proboscis, compared to the body wall, with match against Toxins database. Proteins that have significantly higher expression (logFC > 2) in the proboscis and match in Toxins database, were matched against Pfam, NCBInr customised for Toxins and Swissprot databases. (**Excel**).

Table S2. Primer sequences.

Target	Forward (5'–3')	Reverse (5'–3')	Amplicon Size (bp)
Gapdh	CAGGGGTGCTAAGCAGTTGG	GAGAAGGCTGGGGCTCATTT	165
Crisp	TGTAATGGCTGACGGTCTCG	AGAACATGATGTGGGGCTCG	123
C type lectin	TAGCCATGTCACTCAGCGTC	ACCTGCTCGAATAACGGTGG	130
Hyal	ACGGAGCCATTTCACCCTTT	AGATTTTGAGCAGGCCACGA	137
Reprolysin	GACACTGTGGCCTTACTCCC	TAATAGCTTCGGTCCCGTGC	132
Astacin	TGACCAGGCAATCACAAGGG	CATGATGTTTCTCGCGCCAC	134

Table S3. Translated aminoacid sequences from *Eulalia* used in phylogenetic analyses. (**Excel**)

Table S4. Accession numbers of sequences employed in multi-trait phylogenetics. The model combined CRISP, Hyaluronidase, Astacin, Reprolysin, C type lectin, Serine protease, Endothelin converting enzyme and EGF domain-containing homologs from representative species of key Eumetazoan phyla (venomous and non-venomous organisms). Refer to Figure. 4 in the main article for the full phylogenetic model.

Species	Phylum	CRISP	Hyaluronidase	C Type Lectin	Serine Protease	Astacin	Reprolysin	EGF Domain-Containing Protein	Endothelin Converting Enzyme
Glycera dibranchiata	Annelida	GASB01046798.1	GASB01020098.1	GASB01040405.1	GASB01005460.1	GASB01021908.1	GASB01030463.1	GASB01017903.1	GASB01026619.1
Capitella teleta	Annelida	ELU09214.1	ELT97073.1	ELT92401.1	ELU12131.1	ELT93756.1	ELT98077.1	ELU11161.1	ELT90095.1
Parasteatoda tepidariorum	Chelicerata	XP_015904653.1	XP_015922490.1	XP_021000063.1	XP_015908189.2	XP_015905753.1	XP_015921582.1	XP_015911895.1	XP_015927713.1
Stegodyphus mimosarum	Chelicerata	KFM73511.1	KFM61642.1	KFM65020.1	KFM57227.1	KFM60032.1	KFM61983.1	KFM70600.1	KFM77382.1
Centruroides sculpturatus	Chelicerata	XP_023221739.1	XP_023226974.1	XP_023222872.1	XP_023218618.1	XP_023222430.1	XP_023240235.1	XP_023215803.1	XP_023211934.1
Exaiptasia pallida	Cnidaria	XP_020904389.1	XP_020913987.1	XP_028518136.1	XP_020916782.1	XP_020907018.2	XP_020916248.1	XP_020899053.1	XP_020899185.1
Pocillopora damicornis	Cnidaria	XP_027048055.1	XP_027044971.1	RMX49478.1	RMX51212.1	XP_027055506.1	XP_027050425.1	XP_027051833.1	XP_027046906.1
Apis mellifera	Hymenoptera	XP_016766808.1	NP_001011619.1	XP_397293.3	XP_016769420.1	XP_003250231.1	XP_006562833.2	XP_026295156.1	XP_392043.3
Homo sapiens	Mammalia	AAI01540.1	AAH05896.1	AAA35726.1	XP_011518940.1	AAH13871.1	EAW62382.1	NP_060087.3	NP_001106819.1
Rattus norvegicus	Mammalia	AAD48090.1	NP_997482.2	1TDQ_B	EDL83068.1	EDL94198.1	NP_001101903.1	NP_001099191.1	BAA06152.1
Octopus bimaculoides	Mollusca	XP_014790560.1	XP_014771328.1	KOF89271.1	XP_014787085.1	KOF87573.1	KOF93529.1	XP_014781344.1	XP_014790282.1
Mizuhopecten yessoensis	Mollusca	XP_021366565.1	XP_021353240.1	XP_021341992.1	XP_021351138.1	XP_021365700.1	XP_021341707.1	XP_021342460.1	XP_021353180.1
Pomacea canaliculata	Mollusca	XP_025083739.1	XP_025098740.1	XP_025083615.1	XP_025114564.1	XP_025092025.1	XP_025104189.1	PVD22447.1	XP_025083320.1
Python bivittatus	Reptilia	XP_025028116.1	XP_007420722.1	XP_007422481.1	XP_025032198.1	XP_007434871.1	XP_007422351.1	XP_007426224.1	XP_007441074.1
Paroedura picta	Reptilia	GCF55372.1	GCF53585.1	GCF42045.1	GCF56440.1	GCF44322.1	GCF58184.1	GCF55798.1	GCF49036.1
Danio rerio	Teleostei	XP_021322709.1	NP_001121765.1	XP_002660413.1	NP_001099071.1	AAH95288.1	XP_700384.3	XP_005168706.1	NP_001071260.1
Cyprinus carpio	Teleostei	XP_018974179.1	KTF93957.1	KTG02892.1	KTF77069.1	XP_018918842.1	KTG21912.1	AOW71522.1	KTF75647.1
Carassius auratus	Teleostei	XP_026109449.1	XP_026115374.1	XP_026061246.1	XP_026069014.1	XP_026090900.1	XP_026090266.1	XP_026067988.1	XP_026131351.1
